# Graphene Oxide and Reduced Graphene Oxide Nanoflakes Coated with Glycol Chitosan, Propylene Glycol Alginate, and Polydopamine: Characterization and Cytotoxicity in Human Chondrocytes

**DOI:** 10.3390/nano11082105

**Published:** 2021-08-19

**Authors:** Lorenzo Vannozzi, Enrico Catalano, Madina Telkhozhayeva, Eti Teblum, Alina Yarmolenko, Efrat Shawat Avraham, Rajashree Konar, Gilbert Daniel Nessim, Leonardo Ricotti

**Affiliations:** 1 The BioRobotics Institute, Scuola Superiore Sant’Anna, 56127 Pisa, Italy; enrico.catalano@santannapisa.it; 2 Department of Excellence in Robotics & AI, Scuola Superiore Sant’Anna, 56127 Pisa, Italy; 3 Department of Chemistry and Institute of Nanotechnology, Bar-Ilan University, Ramat Gan 52900, Israel; telkhozhayeva@gmail.com (M.T.); eti.teblum@gmail.com (E.T.); alina.y@outlook.co.il (A.Y.); epale086@gmail.com (E.S.A.); rajashreekonar@gmail.com (R.K.); gilbert.nessim@biu.ac.il (G.D.N.)

**Keywords:** graphene oxide, reduced graphene oxide, human chondrocytes, cytotoxicity, glycol chitosan, propylene glycol alginate, polydopamine, LDH, polydispersity index

## Abstract

Recently, graphene and its derivatives have been extensively investigated for their interesting properties in many biomedical fields, including tissue engineering and regenerative medicine. Nonetheless, graphene oxide (GO) and reduced GO (rGO) are still under investigation for improving their dispersibility in aqueous solutions and their safety in different cell types. This work explores the interaction of GO and rGO with different polymeric dispersants, such as glycol chitosan (GC), propylene glycol alginate (PGA), and polydopamine (PDA), and their effects on human chondrocytes. GO was synthesized using Hummer’s method, followed by a sonication-assisted liquid-phase exfoliation (LPE) process, drying, and thermal reduction to obtain rGO. The flakes of GO and rGO exhibited an average lateral size of 8.8 ± 4.6 and 18.3 ± 8.5 µm, respectively. Their dispersibility and colloidal stability were investigated in the presence of the polymeric surfactants, resulting in an improvement in the suspension stability in terms of average size and polydispersity index over 1 h, in particular for PDA. Furthermore, cytotoxic effects induced by coated and uncoated GO and rGO on human chondrocytes at different concentrations (12.5, 25, 50 and 100 µg/mL) were assessed through LDH assay. Results showed a concentration-dependent response, and the presence of PGA contributed to statistically decreasing the difference in the LDH activity with respect to the control. These results open the way to a potentially safer use of these nanomaterials in the fields of cartilage tissue engineering and regenerative medicine.

## 1. Introduction

Graphene-derived materials have received extensive attention for their exceptional mechanical, electrical, thermal, and optical properties; drug-loading capacity; high surface area-to-volume ratio; and unique atomic and molecular structure [1,2]. Among them, graphene oxide (GO) with numerous functional groups has intriguing properties that encourage its application in many scientific fields, including biomedical ones, in particular tissue engineering and regenerative medicine [3]. Indeed, one of the main advantages of GO is that it can feature several reactive chemical groups on its surface, allowing modification or functionalization. In addition to GO, its reduced form, namely rGO, has been investigated because of its enhanced electrical conductivity caused by a reduction in the GO oxygen content [4]. 

Both materials are generally featured by a nanometric thickness and a large lateral dimension, from hundreds of nanometers up to tens of microns, making them versatile for several applications. For example, GO and rGO have been used as electrical dopant agents in conductive composite materials [5,6], for mechanical reinforcement of polymeric matrices [7], or as lubricant agents to improve the wear of scaffolds to be used as substitutes for load-bearing joints [8,9]. 

Understanding the parameters controlling the dispersibility of GO and rGO and the properties of the as-prepared dispersions is extremely important for implementing these materials in biomedical applications [10,11]. Their colloidal stability and behavior are influenced by many factors, such as particle size, zeta potential, and pH [12]. The interaction between GO electrostatic and hydrophobic π domains provides it with negatively charged domains, regulating its colloidal stability and dispersion in solution. Similarly, rGO is generally characterized by a negative charge, even if lower than GO, because of fewer negative functional groups. Nonetheless, both GO and rGO are affected by re-aggregation phenomena in aqueous solutions due to van der Waals forces’ interactions between non-polar molecules that cause precipitation and agglomeration of the nanomaterials over time. Thus, there is the need to investigate further interactions between single GO and rGO functional groups and dispersant chemical groups to improve their colloidal stability. Indeed, the distinctive π–π stacking interaction provides a large specific surface area for a high loading and absorption capacity to GO and rGO [2]. Recently, several attempts have been made to correlate GO and rGO stability in solution, investigating different parameters, such as dipole moment, surface energy, and Hansen and Hildebrand solubility parameters [13]. For example, Ayán-Varela et al. investigated the dispersibility of rGO in a wide range of solvents, also analyzing the results based on surface energies and Hansen solubility parameters, hypothesizing a rational design of solvent mixtures that surpassed the best single-component solvents [14]. 

The colloidal stability also plays a pivotal role in the GO/rGO interaction with cells [15]. In the scientific literature, the interaction mechanism of graphene derivatives is determined by many factors, including the size, surface charge, and agglomeration rate of the flakes [16]. It is known that a reduced size (in terms of average thickness and lateral size) can promote higher toxic effects on different kinds of cells (NIH 3T3, U87, A549, RAW 264.7, NB4, and HL60 cells) [17]. Regarding the surface charge, some results showed that rGO nanomaterials are less toxic than their respective chemical parental GOs [18]. This was primarily ascribed to the loss of oxygen-containing functional groups that are rather reactive to biomolecules and the intracellular environment [19]. Nonetheless, reports demonstrated that rGO is more toxic than GO for specific cell types, such as alveolar epithelial cells [20,21]. Overall, several studies have suggested dose- and time-dependent cytotoxicity of GO and rGO, which can enter the cytoplasm and nucleus, decrease cell adhesion, and induce apoptosis [22,23]. This correlation would promote the killing of cancer cells (e.g., lung cancer cells) but needs to be further investigated for a safer interaction with other types of cells. For example, Wojtoniszaka et al. investigated the viability of mice fibroblasts (using the cell line L929) [24], observing that GO and rGO significantly decreased cell viability after 48 h at concentrations between 50 μg/mL and 100 μg/mL. On the other hand, GO and rGO at lower concentrations (<50 μg/mL) exhibited a higher cytocompatibility level than the control. To the best of our knowledge, the dose- and time-dependent cytotoxicity of GO and rGO has never been tested on human chondrocytes. The cytotoxic analysis on human chondrocytes may assume relevant importance because of the peculiarity of the articular cavities, which are closed spaces along the peripheral circulation route featured by a slow rate interchange of nutrients with the blood [25]. Using such carbon-based nanomaterials in biodegradable scaffolds for the replacement or regeneration of functions in the cartilage can promote the release of these materials. The knowledge of their effects on chondrocytes can represent a piece of helpful information to avoid undesired effects when choosing the material components and relative concentrations.

In this study, we investigated the dispersion of GO and rGO in water by exploring three different types of dispersants, namely glycol chitosan (GC), propylene glycol alginate (PGA), and polydopamine (PDA). GC is a biocompatible, water-soluble, and amphiphilic chitosan derivative with both hydrophilic ethylene glycol branches and hydrophobic segments, which can self-assemble around hydrophobic materials to make them stable in aqueous environments [15,26]. This material has already been used for building GO-GC conjugates to be included in 3D scaffolds [27]. Recently, PDA, the final oxidation product of dopamine, has attracted attention for its versatility due to several functional groups (such as catechol, amine, and imine) [28]. These groups can act as active sites for modification with desired target materials. Thus, PDA has recently emerged as an interesting material for functionalizing and coating organic and inorganic compounds. While GC has been widely investigated in the literature [29,30], and PDA has recently emerged as a multifunctional and multipurpose polymer, the use of PGA is relatively new for this kind of application. PGA is a stabilizer agent usually used in food products. PGA is a negatively charged ester of alginic acid, which is derived from kelp. Its use for the dispersion of carbon-based nanomaterials has not been investigated yet in the scientific literature.

In this paper, we investigate for the first time the colloidal stability of dispersions obtained by applying the mentioned polymers and the cytotoxicity of GO and rGO coated with these molecules toward human chondrocytes for up to 48 h. 

## 2. Materials and Methods 

### 2.1. Synthesis of Graphene Oxide and Reduced Graphene Oxide

Graphite oxide was synthesized by oxidation of graphite powder according to Hummer’s method [31]. Concentrated sulfuric acid (H_2_SO_4_, 184 mL) was added to a mixture of graphite powder (4.0 g, 1 wt. equiv) and sodium nitrate (NaNO_3_, 4.0 g, 0.5 wt. equiv), and the mixture was cooled in an ice bath. After waiting for 30 min until the solution reached room temperature, potassium permanganate (KMnO_4_, 12.0 g, 3 wt. equiv) was slowly added, keeping the reaction at room temperature. First, the solution was stirred for 2 h. Then, deionized water was slowly added (184 mL), and the mixture was kept in ice). Next, 30% hydrogen peroxide (H_2_O_2_, 18 mL) was carefully added to the solution. The mixture was centrifuged (12,000 rpm for 1 h) and washed with distilled water 3 times for workup. Next, the mixture was centrifuged (12,000 rpm for 1 h) and washed with 5% hydrochloric acid (HCl) solution 2 times. Then, the mixture was extensively rewashed with distilled water and centrifuged (12,000 rpm for 1 h) until the solution reached pH = 7, and finally was dried in a vacuum furnace for 24 h. Finally, the obtained graphite oxide (brownish solid) was exfoliated to GO flakes after 2 h of sonication-assisted liquid-phase exfoliation (LPE) (Elmasonic P, bath frequency: 80 kHz, 100 W) [32]. 

Chemical vapor deposition (CVD) was used to reduce GO to rGO (black solid) [32]. Dried GO was loaded into a quartz tube and inserted into a Lindberg Blue furnace preheated to 900 °C for 2 h, and reduced by hydrogen flow (400 sccm) under an argon (100 sccm) atmosphere.

Next, GO and rGO were autoclaved through vapor steam (30 min at 121 °C) to ensure their sterilization before their use for cytocompatibility tests according to ISO standard 17665-1:2006.

### 2.2. Chemical and Morphological Characterization

Elemental analysis was performed using the Thermo CHNS-O elemental analysis device, model EA 1110. 

Raman spectroscopy was conducted using a Raman spectrometer (ONDAX, XLF-MICRO, Monrovia, USA) with a 532 nm excitation laser. GO and rGO flakes were deposited on Si/SiO_2_ by drop-casting of one droplet of GO and rGO previously dispersed in ethanol (after 1 h sonication) and dried overnight.

Atomic force microscopy (AFM) measurements were carried out using a Bio FastScan scanning probe microscope (Bruker, Dimension Icon & FastScan Bio, Karlsruhe, Germany). All images were obtained using PeakForce Quantitative Nanomechanical Mapping mode with a Fast Scan C (Bruker) silicon probe (spring constant: 0.45 N/m). The microscope was covered with an acoustic hood to minimize vibrational noise. The measurements were performed under environmental conditions. The images were captured in the retrace direction with a scan rate of 1.6 Hz. The resolution of the images was 512 samples/line. For image processing and thickness analysis, NanoScope Analysis software was used. Before thickness analysis, images underwent “flatting” and “planefit” functions. GO and rGO flakes were deposited on Si/SiO_2_ wafers, as prepared for Raman spectroscopy. A detailed AFM statistical analysis on a large number of GO and rGO flakes (30 flakes for each sample) after sonication (80 kHz, 100 W) in ethanol was performed to obtain data on their lateral size and thickness distribution.

### 2.3. Nanomaterial Coating 

The stability of GO and rGO dispersions in aqueous solution was tested by applying three types of polymers: glycol chitosan (GC), propylene glycol alginate (PGA), and polydopamine (PDA). 

GC (degree of polymerization > 400, Sigma-Aldrich, St. Louis, MO, USA) and PGA (degree of esterification < 80%, Carbosynth, Staad, St. Gallen, Switzerland) solutions were prepared at a concentration of 1 mg/mL in deionized water and then filtrated (filter size: 0.22 μm). Sterilized GO or rGO was added in a ratio of 1:1 *w/w* to the polymeric solutions. Then, a sonication process with an ultrasound probe (power: 25 W, time: 300 s, frequency: 20 kHz, Bandelin SonoPuls HD4050, Berlin, Germany) was performed to enhance the interaction between the polymer and the GO/rGO, favoring the dispersion of nanomaterials. 

The modification of GO/rGO with PDA was performed according to the protocol published in [33]. First, GO/rGO (5 mg/mL) was suspended in deionized water. Then, the pH of the GO/rGO dispersion was adjusted by the drop addition of Tris-buffer HCl solution at a concentration of 0.1 M (Sigma-Aldrich) to achieve a pH of 8.5. Next, the solution was sonicated with an ultrasound probe (power: 25 W, time: 300 s, frequency: 20 kHz). Then, a filtrated solution (filter size: 0.22 μm) of dopamine hydrochloride (Sigma-Aldrich) (5 mg/mL) was added in a ratio of 3:2 to the GO/rGO dispersion, and the mixture was stirred vigorously for 24 h at room temperature. 

### 2.4. Dispersion Analysis

Dynamic light scattering (DLS) was used to evaluate the stability of the nanomaterial dispersions over time and their zeta potential. DLS and zeta potential measurements were performed using Zetasizer NanoZS90 (Malvern Instruments Ltd., Worcestershire, UK), analyzing the average size and polydispersity index (PDI) immediately after sonication over 1 h, analyzing these parameters every 10 min. The samples were dispersed in water for DLS and ethanol (neutral charge) for the zeta potential, and the concentration of GO and rGO for all sample types was set to 100 μg/mL, the concentration set for the optimal measurement results for DLS and the zeta potential. The average size at each specific time point represents the mean ± standard deviation of three different samples. 

### 2.5. XPS Analysis

X-ray photoelectron spectroscopy (XPS) analysis was carried out using a Nexsa spectrometer (Thermo Scientific, Sunnyvale, USA) equipped with a monochromatic, micro-focused, low-power Al Ka X-ray source (photon energy: 1486.6 eV). High-resolution spectra were acquired at a pass energy of 50 eV. The source power was typically 72 W. The measurements had been carried out under ultra-high-vacuum conditions, at a base pressure of 5 × 10^−10^ torr (and no higher than 3 × 10^−9^ torr). The obtained spectra were analyzed and deconvoluted using Vision software (Kratos). Overlapping signals were analyzed after deconvolution into Gaussian/Lorentzian-shaped components.

### 2.6. Cytotoxic Assay

Human articular chondrocytes (Cell Applications Inc., Boston, MA, USA) were cultured in chondrocyte growth medium (Cell Applications Inc., Boston, MA, USA) in 75 cm^2^ flasks. Chondrocytes used for the experiments were at passage numbers lower than 5. For cell culture experiments, GO and rGO dispersions (bare and with dispersants) at different concentrations (12.5 μg/mL, 25 μg/mL, 50 μg/mL, and 100 μg/mL) were prepared from stock dispersions (1 mg/mL) after sonication by diluting each dispersion with the cell culture medium. Then, the dispersions were added to a 48-well plate after 24 h from cell seeding, in which human chondrocytes were previously seeded at a density of 5000 cells/well. Culture cell supernatants (25 μL) after 24 h and 48 h from the cell exposure to GO/rGO were collected and transferred into a new 96-well microplate, and the Lactate Dehydrogenase Activity Assay Kit (Sigma-Aldrich) was used according to the manufacturer’s instructions. This kit quantitatively measures lactate dehydrogenase (LDH) activity, which can be used as an indicator of membrane integrity. In this kit, LDH reduces NAD to NADH, specifically detected by colorimetric (450 nm) assay. In negative control wells, the cells were incubated in culture media alone. Three independent samples were analyzed for each sample type and for each time point. 

### 2.7. Statistical Analysis

All experimental data derived from DLS analysis and LDH assay were statistically analyzed with Kruskal–Wallis one-way ANOVA and Dunn’s post hoc tests (GraphPad Prism 8) to analyze significant differences between groups. The significance level was set at 5%. 

## 3. Results and Discussion 

### 3.1. GO and rGO Characterization

The composition of synthesized bulk GO and rGO was investigated using atomic absorption spectroscopy (Table 1) to examine the degree of reduction of GO after heating it in the presence of H_2_ at 900 °C [34]. 

The results confirmed that the reduction was successful, as the %O value dropped from 50.21 ± 0.10 (GO) to 6.26 ± 0.26 (rGO). 

Detailed AFM statistical analysis of exfoliated GO and rGO flakes was performed to analyze the distribution of the lateral size and thickness of the synthesized flakes throughout the wafer. Bath sonication at 80 kHz frequency (100 W) was used to form thinner and larger lateral size flakes, applying a technique based on sonication-assisted LPE recently proposed by our group [32].

**Figure 1:**
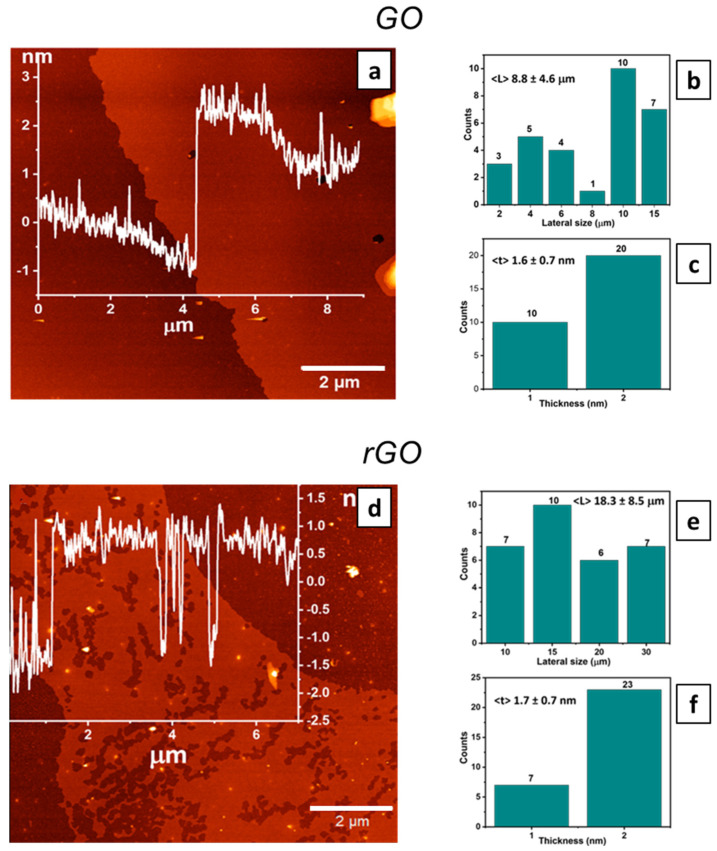
(**a**) Representative AFM image with a cross-sectional profile; average dimensional analyses of (**b**) the lateral size and (**c**) thickness distribution of GO flakes in ethanol dispersions. (**d**) Representative AFM image with a cross-sectional profile; average dimensional analyses of (**e**) the lateral size and (**f**) thickness distribution of rGO flakes in ethanol dispersions.

The cross-sectional profile thickness of GO flakes was 1.5 ± 0.3 nm (Figure 1a), while the average lateral size was 8.8 ± 4.6 μm for GO flakes (Figure 1b). Additionally, the average thickness of GO flakes was measured as 1.6 ± 0.7 nm (Figure 1c). Regarding the rGO flakes, the cross-sectional profile thickness was 1.8 ± 0.3 nm (Figure 1d) and the average lateral size was 18.3 ± 8.5 μm (Figure 1e). The average thickness was 1.7 ± 0.7 nm (Figure 1f). The presence of some defects (holes) detected on the surface of the rGO flakes might be ascribed to the reduction process at high temperatures (Figure 1d). 

**Figure 2:**
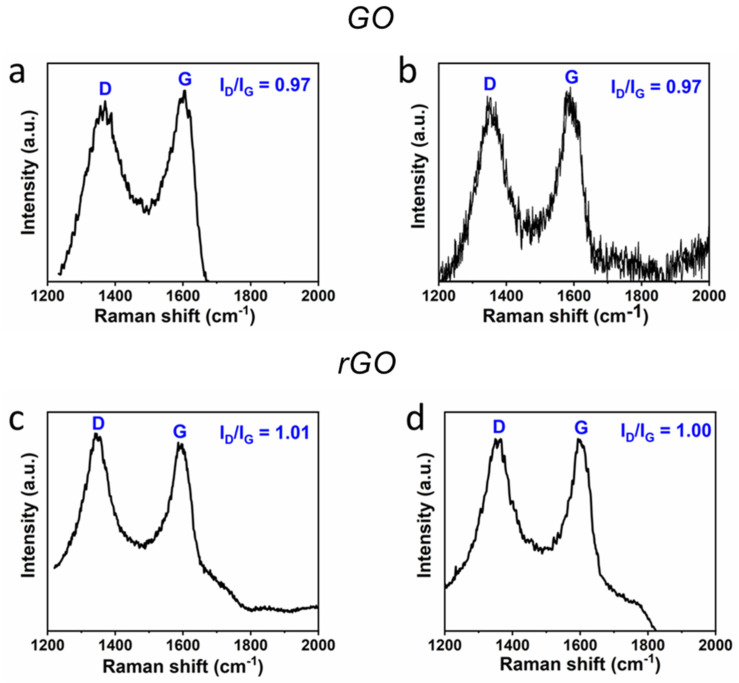
Raman spectra of GO flakes on Si/SiO_2_ before (**a**) sterilization and after (**b**) sterilization in an autoclave. Raman spectra of rGO flakes on Si/SiO_2_ before (**c**) sterilization and after (**d**) sterilization in an autoclave.

In addition to elemental analysis, Raman spectroscopy is generally used to characterize crystal structure, disorder, and defects in graphene-based materials. The GO and rGO spectra (Figure 2a,c) showed two well-defined peaks, D and G, at 1355 and 1590 cm^−1^, respectively. The G peak is due to C=C stretching vibrations in graphene planes of the cluster, whereas the D peak is attributed to a breathing mode of A1g symmetry, which is considered absent in perfect crystalline graphite and becomes active only when it is disordered [35]. Raman spectroscopy was also performed to analyze the crystal structure of autoclaved GO and rGO to verify the absence of modification after the sterilization treatment (Figure 2b,d). Results confirmed the absence of significant chemical changes in the nanomaterial structures after the sterilization process. Indeed, the I_G_/I_D_ ratio remained similar for GO and rGO compared to the values before sterilization. 

### 3.2. Nanomaterial Coating

**Figure 3:**
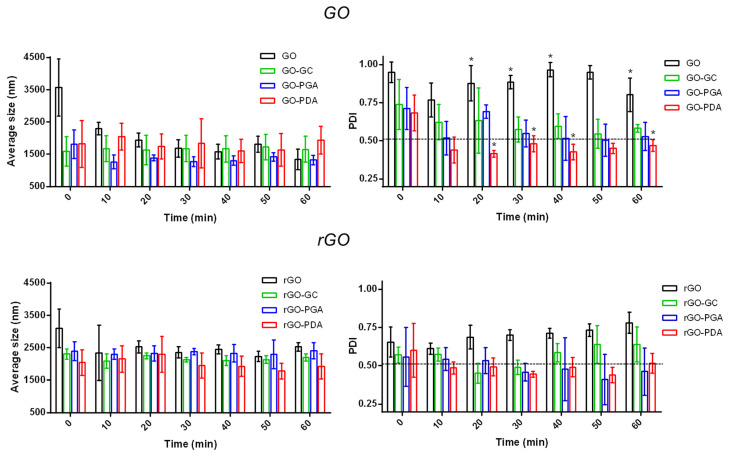
Dynamic light scattering analysis of GO (**top**) and rGO (**bottom**) samples (with and without coatings) in terms of average size (left) and polydispersity index (PDI, right). The dashed line represents a maximum “acceptance threshold” set at 0.5 [15] for the PDI. GO, uncoated graphene oxide (control); GO-GC, graphene oxide coated with glycol chitosan; GO-PGA, graphene oxide coated with propylene glycol alginate; GO-PDA, graphene oxide functionalized with polydopamine; rGO, uncoated reduced graphene oxide (control); rGO-GC, reduced graphene oxide coated with glycol chitosan; rGO-PGA, reduced graphene oxide coated with propylene glycol alginate; rGO-PDA, reduced graphene oxide functionalized with polydopamine. *N* = 3. * = *p*-value < 0.05.

The stability of GO and rGO was assessed by evaluating the steadiness of the average size and the PDI over time. Figure 3 shows the results achieved for the GO and rGO samples provided with different coatings compared to uncoated materials. 

The GO samples showed an average size below 2000 nm for all types of surfactants used. The control (GO) qualitatively did not show a regular PDI value over time, which was far from the threshold value set at 0.5. Such instability may be due to the tendency of GO flakes to aggregate without the presence of a stabilizing agent. In particular, PDA resulted in the most effective coating for GO, providing an almost constant average size value and a PDI below 0.5 over time, and statistically different than the uncoated GO. 

The rGO samples had an average size of 2000–2500 nm for all types of surfactants used in the procedure of polymer wrapping. In addition, the uncoated material had a relatively high PDI, highlighting the difficulty of obtaining a stable dispersion without using a surfactant. In this case, all the polymers (GC, PGA, and PDA) guaranteed the achievement of a stable average size, but only the use of PGA and PDA ensured a PDI value stably below 0.5. 

It is worth mentioning that DLS allows analyzing the stability of suspensions. However, it relies on the assumption of spherical particles. For this reason, the average size of GO and rGO was lower than what was declared in Section 3.1, probably caused by the flakes being wrapped in the aqueous solutions. Furthermore, a relatively high PDI value compared to the acceptance threshold can also be considered acceptable in the case of non-spherical nanomaterials, like in our case [36]. 

The analysis of the zeta potential of all material formulations is reported in Table 2.

The zeta potential is considered a key indicator for the stability of a solution. It may include electrostatic repulsion or steric stabilization by incorporating a polymer at the surface either by adsorption from solution or by chemically attaching something to the surface particles [37]. Indeed, suspensions can be stable due to inertial effects caused mainly by the polymer coating (van der Waals forces). In that case, the zeta potential may be closer to zero [38]. It is well-known that GO flakes in polar solvents, such as water or ethanol, are negatively charged because of the presence of ionized carboxylic acid groups (-COO^−^) [39], while the charge of rGO is less negative due to the lower oxygen content. In our case, the wide difference between GO and rGO in the oxygen content showed in Table 2 justified the different values (−13.60 and 0.77, respectively) reported in Table 2. Furthermore, the application of polymeric surfactants affected the zeta potential of GO and rGO flakes in ethanol (neutral pH), demonstrating an effective modification of their surface charge. In general, GO and rGO flakes are featured by an amphiphilic nature with an edge-to-center distribution of hydrophilic and hydrophobic domains, promoting interfaces to lower the total interfacial energy toward forming as minimal an amount of large precipitates as possible. Thus, the evaluation of the possible modifications in the zeta potential value can further highlight potential interactions with the polymeric surfactants. More in detail, GC and PDA tended to make slightly more positive the GO, probably due to the higher presence of amine groups. However, these polymers provoked a decrease in the zeta potential of rGO, probably because of the intrinsic amphoteric nature of GC and the interaction with several functional groups owned by PDA (e.g., alkyl, carboxyl, carboxyl derivative groups, amino, azido), which are not only positive. Lastly, PGA likely promoted the dispersion, thanks to its steric stabilization with its higher molecular weight with respect to the other polymers. 

**Figure 4:**
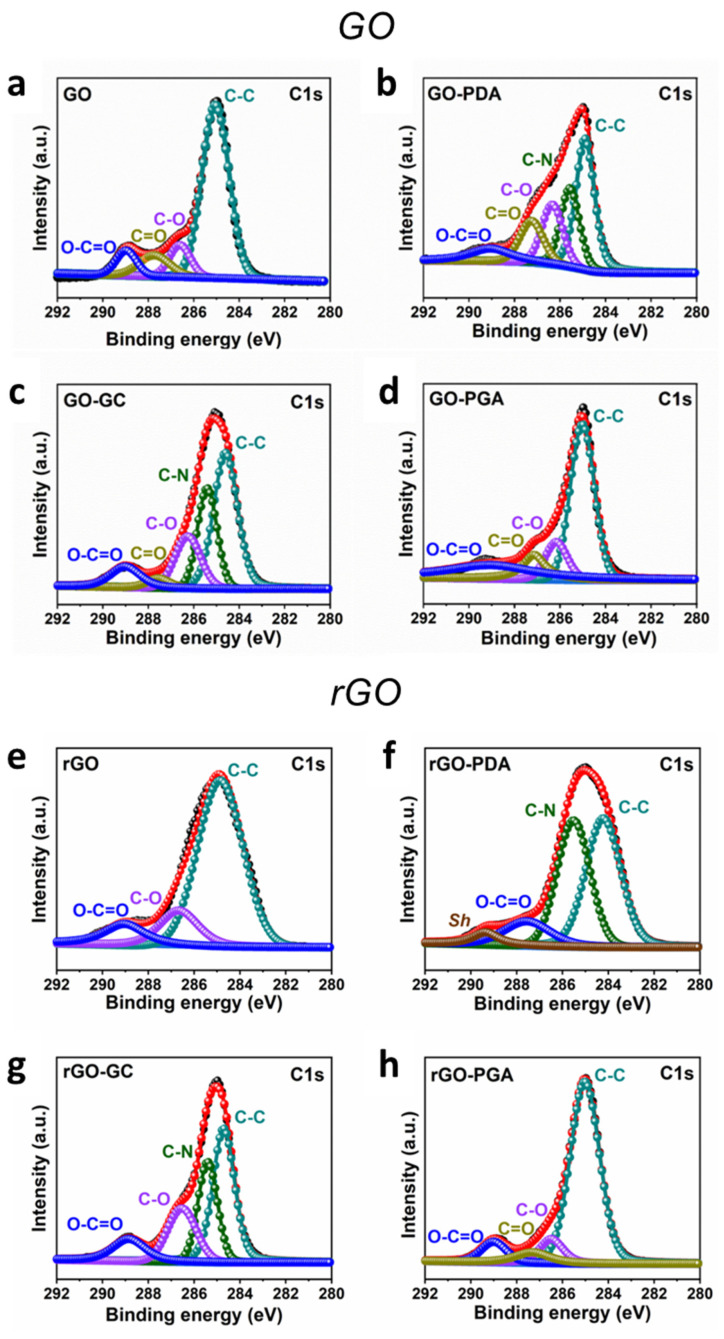
High-resolution XPS C1s spectra of GO (**a**), GO-PDA (**b**), GO-GC (**c**), and GO-PGA (**d**) and high-resolution XPS C1s spectra of rGO (**e**), rGO-PDA (**f**), rGO-GC (**g**), and rGO-PGA (**h**).

Following the zeta potential analysis, XPS was performed to confirm further the polymers’ interactions and attachments with GO and rGO. Figure 4 shows the high-resolution XPS spectra of the C1s region of GO and rGO and their functionalized forms, respectively.

The deconvoluted C1s profile of pristine GO (Figure 4a) shows complex surface chemistry corresponding to the presence of C–C (non-oxygenated C rings, 284.98 eV), C–O (ether, 286.68 eV), C=O (ketone, 287.68 eV), and O–C=O (carboxyl, 288.98 eV), as expected for a GO surface [40]. The XPS C1s spectrum of the GO-PDA system (Figure 4b) can be curve-fitted into five peak branches. The peak at ~284.88 eV is correlated with sp^2^-hybridized carbon atoms arranged in aromatic rings, with no bonding to heteroatoms. The carbon–nitrogen bonds on amines and imines are related to the peak centered at ~285.56 eV. However, it is almost impossible to distinguish primary from secondary amines and specific amino groups due to their close values of the binding energies. Nonetheless, nitrogen atoms are usually present in polydopamine as secondary amines (C–NH–C) and/or imines (C–N=C↔C=N–C). In addition, the C–O bond gave rise to the peak at 286.33 eV. The binding energy of the peak at ~287.23 eV is assigned to quinone or ketone groups (C=O). Moreover, a peak at 289.23 eV results from the O–C=O group [41]. Therefore, we can assume that the presence of the C–N peak suggests that PDA is coated on the surface of GO. Similarly, the C1s profile of the GO-GC system (Figure 4c) can be divided into five fitted curves at binding energies centered at 284.58, 285.38, 286.28, 287.58, and 289.08 eV, which correspond to C–C, C–N, C–O–C, C=O, and O–C=O, respectively [42]. The existence of C–N groups is due to the interaction between –NH_2_ groups of chitosan and the surface of GO. The C1s spectrum of GO-PGA (Figure 4d) has a similar profile with pristine GO. The intensity ratio of C/O is 2.44 in the GO spectrum, and the intensity ratio of C/O is 2.19 in the GO-PGA spectrum. This slight difference in the intensity ratios implies that the amount of oxygen in GO-PGA is more than that in GO, which exists in the functional groups. 

The C1s spectrum of rGO (Figure 4e) shows a significant peak at 284.86 eV, corresponding to graphitic carbon. The shoulder peak at 286.66 eV corresponds to the C–O bond. In addition, there is a complete loss of the C=O peak in the C1s data (287.68 eV) [43], confirming the reduction of GO after heating in the presence of H_2_. In addition, the peak position of the carboxylate carbon (O–C=O) was slightly shifted compared to bare GO. For the C1s XPS spectrum of rGO-PDA (Figure 4f), the main peak at 284.18 eV corresponds to C–C, while the other two components at 285.58 and 287.54 correspond to C–N and O–C=O, respectively. An additional shake-up satellite peak, *Sh*, at 289.34 eV is assigned to the π conjugated system of the aromatic rings of PDA, which suggests that π–π stacking might be one of the possible interactions of PDA and the surface of rGO [44]. Figure 4g shows the XPS peaks of the C1s region for rGO functionalized with GC. The peak at 284.68 eV is mainly due to the contribution of C–C. The other peaks at 285.40, 286.58, and 288.84 eV are ascribed to the functional groups of C–N, C–O, and O–C=O, respectively. The arisen functional group of C–N may be due to the attachment of the GC chains [45]. Finally, Figure 4h presents high-resolution XPS C 1s spectra of rGO-PGA. The peak at ~287.3 eV demonstrates additional oxygen-containing groups (C=O); in contrast, it is absent in bare rGO. 

The physicochemical properties of nanomaterial surfaces play a crucial role in determining the interaction with biomolecules. Previous studies have shown that chemical interactions at the GO/solvent interface are of primary importance, for example, the strong hydrogen bonding established between GO functional groups and solvent molecules [10]. The choice of the dispersant would depend on the end use of GO and rGO, and finding optimal functionalization by ionic and non-ionic surfactants is still challenging, as targeting PDI values below 0.5 are difficult to achieve [46,47]. Noncovalent interaction represents one of the most affordable strategies to modify the surface chemistry of GO and rGO. This mechanism involves weak van der Waals forces and hydrophobic, electrostatic, and π–π stacking interactions, usually promoted after direct sonication [48]. The noncovalent bonds may vary depending on the surface properties of GO and rGO and by their morphology and hydrophobicity. Furthermore, the surface charge is a possible critical factor in determining the cytotoxicity of GO and rGO. The presence of oxygen-containing groups in GO results in higher surface energy than rGO, which is more hydrophobic due to its loss of surface polarity [49]. Other types of dispersants have been tested with GO and rGO in recent years, such as polyethylene glycol (PEG), Pluronic P123, or sodium deoxycholate, resulting in a slight improvement in the dispersion stability but guaranteeing a low biocompatible concentration [24]. Alginic acid is another biocompatible dispersant used for the dispersion of graphene-derived materials, even if its biocompatibility has not been tested with GO or rGO [50]. No insights are reported in the state of the art regarding the use of GC and PGA for both GO and rGO, specifically. Thus, our results show for the first time their potential use as surfactants for these carbon-based nanomaterials. In contrast, PDA has been recently used to facilitate the dispersion of these nanomaterials, showing good results in improving their hydrophilic behavior [51]. Our comparative analysis suggests that PGA and PDA represent good candidates as potential surfactants for the dispersion of GO and rGO in aqueous solutions, showing slightly better performance than GC in analyzing the average size. 

### 3.3. Cytotoxicity Analysis

**Figure 5:**
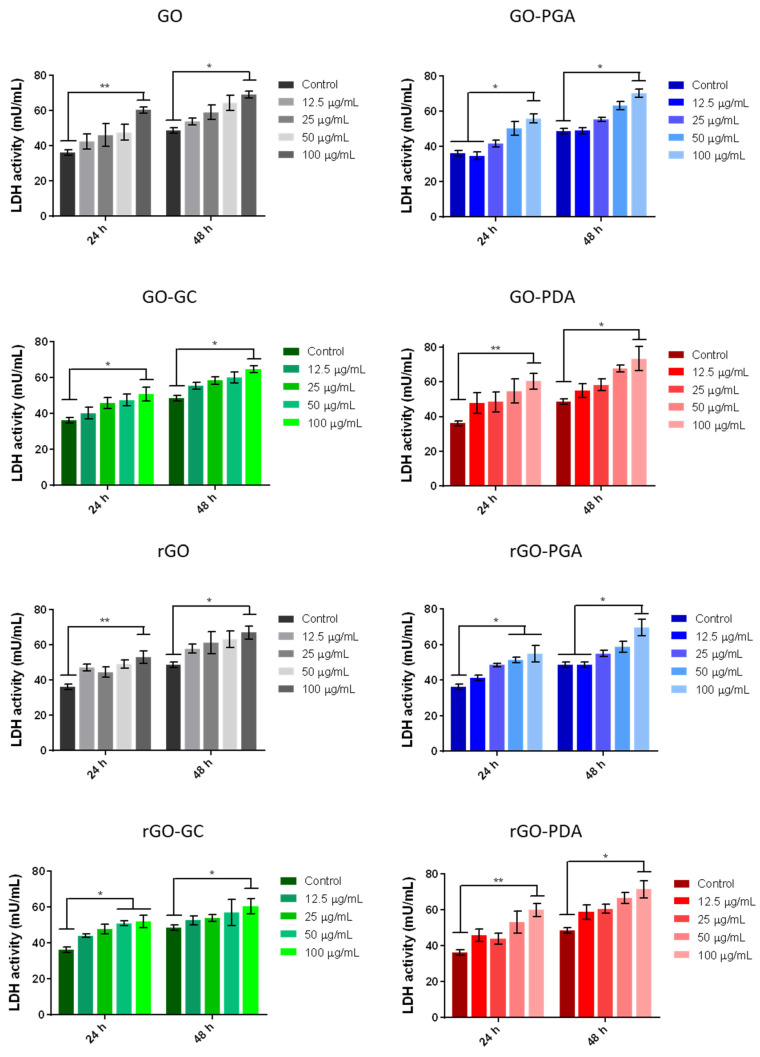
LDH assay of human chondrocytes of GO and rGO flakes with concentrations from 12.5 μg/mL to 100 μg/mL, compared with the use of different polymeric surfactants, namely propylene glycol alginate (GO-PGA and rGO-PGA), glycol chitosan (GO-GC and rGO-GC), and polydopamine (GO-PDA and rGO-PDA). Statistical significance was determined using the Kruskal–Wallis test with Dunn’s post hoc test to validate statistical significance, with * (*p*-value < 0.05) and ** (*p*-value < 0.01).

The cytocompatibility of GO and rGO flakes was assessed on human chondrocytes 24 h and 48 h after exposure. The results are shown in Figure 5. 

Results showed qualitatively dose-dependent LDH activity in human chondrocytes after 24 h and 48 h. The addition of GC and PGA for coating the materials slightly modified the effective LDH activity with respect to the control cases. Their effect was mainly evident after 24 h at a higher concentration of the GO tested (100 µg/mL), for which GC and PGA showed a generally lower release of LDH (*p*-value =< 0.05) and better performance than PDA and uncoated GO (*p*-value =< 0.01). Interestingly, PGA was also effective in keeping the average value of LDH release almost equal to the control for the lowest concentration of GO tested (12.5 µg/mL). After 48 h, the effects of adding the polymeric surfactants were less evident, without any statistical difference. 

Previous studies have revealed that GO with a concentration lower than 20 µg/mL does not exhibit toxic effects on fibroblasts and A549 cells [52,53]. However, GO shows no cytotoxicity on SYS5 cells up to 80 µg/mL [54]. These insights highlight the importance of analyzing the cytotoxic concentration, depending on the cell type. Moreover, our results underline the potential role of using polymeric coatings (especially PGA) to limit the impact of GO on LDH release by human chondrocytes.

The analysis of the effects of rGO also revealed qualitatively dose-dependent LDH activity in human chondrocytes after 24 h and 48 h. In particular, rGO without a coating and coated with PDA showed similar behavior for the highest concentration tested (100 µg/mL) at 24 h and 48 h. Interestingly, a lower significant difference at a concentration of 100 and 50 µg/mL (*p*-value < 0.05) was found when analyzing GC and PGA with respect to uncoated rGO at 24 h. After 48 h, the LDH release trend was similar for all cases, except for the lowest PGA concentration that effectively assisted in keeping the LDH release similar to the control for the lowest concentration of GO tested (12.5 µg/mL). 

Literature evidence shows that GO and rGO are relatively cytocompatible when introduced within 3D scaffolds, such as epithelial cells and fibroblasts [55]. A peculiar aspect of GO is its usefulness in carrying growth factors, such as transforming growth factor β3, which is important to guide mesenchymal stem cells to differentiate into chondrocytes [56]. Controlling the distribution of GO flakes in a 3D environment could contribute to a safer application of those nanomaterials for improving the chondrogenic differentiation of stem cells (concentration up to 600 µg/mL). In contrast, the analysis of nanomaterials directly within the cell culture medium implies a direct contact between adhered cells and the material, which determines cell-related effects even at a lower concentration of materials than in the 3D environment. 

LDH represents an indicator of irreversible cell death due to cell membrane damage [57]. In the literature, a few analyses of LDH release have been performed on both GO and rGO. A study on H9C2 rat myocardial cells showed an increasing level of LDH while testing higher concentrations for rGO with respect to GO. It is relevant to declare that the average size of GO and rGO was in the order of 200 nm [58]. In another study, a better performance was shown by rGO with respect to GO when dealing with the same size (0.4–0.8 µm). [59]. The level of LDH was found to statistically increase even at a low concentration of GO (2.5 µg/mL) in GO with a lateral size that was less than 500 nm [60]. Thus, a comparative analysis between GO and rGO can depend on the nanomaterial size and tested cell type. In general, the toxicity of GO and rGO can be influenced by several factors, such as concentration, oxygen content, lateral size or thickness, and surface charge. It has been suggested that a higher oxygen content in GO is responsible for a safer interaction with cells and possible beneficial effects for cell adhesion and growth [61]. A reduction in the degree of GO oxidation may result in rapid immune cell infiltration and uptake, provoking possible damages to cells [62].

Regarding thickness, several observations have revealed the less toxic behavior of micro-size GO than nano-size GO on embryonic fibroblast cells at high concentrations (100 and 200 µg/mL), confirming the size-dependent toxicity of GO [63]. In fact, smaller GO (less than 300 nm) may be internalized by cells, resulting in higher toxicity than GO at a higher average size (close to 1000 nm) [17]. An investigation of the toxicity of GO in relation to its size was also proposed by Jia et al. [64]. Indeed, size is extremely important because of a possible internalization of GO within the cells, inducing specific response pathways. Their results proved on HEK 293 T cells that small-size nanoplatelets (average diameter < 300 nm) show higher toxicity in terms of cell viability and DNA damage. 

Our analysis highlights the potential role of polymeric dispersants in promoting the dispersion of nano-size GO and rGO in aqueous solution, maintaining high safety for human chondrocytes. Other types of dispersants have been tested with GO and rGO in recent years, such as polyethylene glycol (PEG), Pluronic P123, or sodium deoxycholate [24]. Among them, PEG shows the most cytocompatible properties with mice fibroblast cells (line L929) and higher cell viability (~80%) for a concentration up to 25 µg/mL compared to 50 µg/mL (~60%) and 100 µg/mL (~40%). Katsumiti et al. studied the interaction of GO (0.6–1.2 nm thickness, lateral dimensions between 0.5 and 2 μm, and 20% wt. oxygen content), also stabilized with polyvinylpyrrolidone (PVP), with mussel hemocytes in vitro, finding concentration-dependent toxicity [65]. Furthermore, the authors found that rGO is more toxic than GO, reporting an LC50 value of 43.72 µg/mL against a value of 29.90 µg/mL showed by rGO. The use of PVP supported the dispersion of both nanomaterials, even if it increased the cytotoxicity of GO and rGO because of accumulation in cells. Among the polymers analyzed in our study, PGA represented the best polymeric dispersant, considering GO and rGO dispersion and cytotoxicity results. Indeed, GC can increase cell membrane permeability [66], arising a possible enhanced release of LDH. However, PDA may also induce an increase in LDH activity due to its affinity to iron ions, as recently reported [67]. Future efforts may focus on different aspects of the cell response when exposed to these nanomaterials (e.g., metabolic activity or production of reactive oxygen species), as well as in vivo toxicity, in appropriate animal models.

## 4. Conclusions

We reported the synthesis, dispersion, physicochemical characterization, and cytocompatibility evaluation of GO and rGO flakes on human chondrocytes. GO and rGO, once synthesized, underwent autoclaving for sterilization, and results confirmed the absence of significant chemical changes in the nanomaterial structures after the sterilization process. Both materials were dispersed in deionized water by adding polymeric surfactants, such as GC, PGA, and PDA, and an improvement in the stability of the suspensions was verified with respect to the control. GO and rGO showed LDH release proportional to the concentration, and the use of polymeric surfactants led to different cytotoxicity induced on cells, particularly at the highest concentration (100 µg/mL). Overall, PGA showed the best performance, considering the LDH result for both GO and rGO, while PDA mainly contributed to improving dispersion. 

This study provides further insights into the use of GO and rGO nanomaterials and their cytotoxic effects on human chondrocytes for their possible exploitation in the field of cartilage tissue engineering. Indeed, in this field, the investigation of composite hydrogels/scaffolds embedding carbon-based nanofillers represents a recent trend, with GO and rGO being lubricant agents as well as mechanical reinforcing agents. Nonetheless, a full comprehension of the possible toxic effects of GO and rGO is necessary for the future development of safe composite materials for cartilage regeneration or substitution. 

## Figures and Tables

**Table 1:** Elemental analysis (average % and standard deviation of carbon, oxygen, and hydrogen) for GO and rGO samples.

	% C	% O	% H
GO	46.59 ± 0.20	50.21 ± 0.10	3.06 ± 0.02
rGO	92.60 ± 0.20	6.26 ± 0.26	0.95 ± 0.05

**Table 2:** Zeta potential values measured for GO and rGO in ethanol, with and without coatings.

	No Coating	GC	PGA	PDA
GO	−13.60 ± 0.05	−10.40 ± 1.26	4.73 ± 0.17	−5.04 ± 0.80
rGO	0.77 ± 1.43	−16.60 ± 2.75	−5.55 ± 1.28	−22.80 ± 0.60

## Data Availability

The data presented in this study are available on request from the corresponding author.
